# Relationship of insight to neurocognitive function and risk of recurrence in depression: A naturalistic follow-up study

**DOI:** 10.3389/fpsyt.2023.1084993

**Published:** 2023-03-16

**Authors:** Min Wang, Qiong Liu, Xiao Yang, Yikai Dou, Yu Wang, Zijian Zhang, Ruiqing Luo, Yangrui Ma, Qiang Wang, Tao Li, Xiaohong Ma

**Affiliations:** ^1^Psychiatric Laboratory and Mental Health Center, The State Key Laboratory of Biotherapy, West China Hospital of Sichuan University, Chengdu, China; ^2^Huaxi Brain Research Center, West China Hospital of Sichuan University, Chengdu, China; ^3^Golden Apple Jincheng No.1 Secondary School, Chengdu, China

**Keywords:** major depressive disorder, insight, neurocognitive function, cognitive flexibility, recurrence

## Abstract

**Introduction:**

Major depressive disorder (MDD) is a highly recurrent mental illness accompanied by impairment of neurocognitive function. Lack of insight may affect patients’ motivation to seek treatment, resulting in poor clinical outcomes. This study explores the relationship of insight to neurocognitive function and the risk of recurrence of depressive episodes in patients with MDD.

**Methods:**

Demographic, clinical variables, and neurocognitive function measured with Intra-Extra Dimensional Set Shift (IED) from the Cambridge Neuropsychological Test Automated Battery (CANTAB) were collected from 277 patients with MDD. Among them, 141 participants completed a follow-up visit within 1–5 years. Insight was measured using the 17-item Hamilton Depression Rating Scale (HAM-D). To explore the factors associated with recurrence, binary logistic regression models were used.

**Results:**

Patients with MDD, without insight, had significantly higher total and factor scores (anxiety/somatization, weight, retardation, and sleep) on the HAM-D and worse performance in the neurocognition task, compared to those with insight. Furthermore, binary logistic regression revealed that insight and retardation can predict recurrence.

**Conclusion:**

Lack of insight is associated with recurrence and impaired cognitive flexibility in patients with MDD.

## 1. Introduction

Major depressive disorder (MDD) is a common, recurrent, disabling mental disorder characterized by depressed mood, anhedonia, psychomotor retardation, guilt about being ill, and suicidal tendencies (1). MDD is prevalent in 15–18% of the population, indicating that almost one in five people globally have experienced a depressive episode in their lifetime (2). MDD has been ranked as the third leading cause of disease burden worldwide, and the economic burden continues to increase over time as this population trends undesirable outcomes (3). This implies that considerable effort should be made to identify reliable factors affecting outcomes, so targeted prevention can be implemented.

Recurrence is a serious social problem that not only influences daily life and increases the risk of suicide in depressed individuals but also aggravates the economic burden of families (4). Approximately 50% of those who recover from a first episode of depression experience at least one additional episode throughout their lives, and the risk increases further with each new episode (5). Several putative risk factors have been identified for the recurrence of MDD. Greater severity of preceding depressive episodes, a larger number of previous episodes, higher suicidal ideation in the current episode, presence of residual symptoms, earlier age of onset, younger age at first treatment, and longer duration of untreated illness may predict subsequent depression relapse (6–9). Nonetheless, the evidence of other factors derived from demographic features such as family history, poor social support, lower socioeconomic status, and stressful life events is also limited because of methodological difficulties (4, 5, 10). Despite the nature and treatment of depression being researched for over half a century, we are still incapable of confidently predicting who will experience a recurrence following treatment termination.

Insight is a common concept in psychiatry and broadly refers to the awareness of the illness, the ability to recognize the symptoms, and realize the need for treatment (11). Many studies have examined insight using the single rating item, “Lack of Judgment and Insight (item 12)” from the Positive and Negative Syndrome Scale (PANSS) or “Insight (item 17)” from the Hamilton Depression Rating Scale (HAM-D) (12). As the insight measures become more comprehensive, structured, reliable, and valid, the Scale to Assess Unawareness of Mental Disorder, the Schedule for the Assessment of Insight, and the Treatment Attitudes Questionnaire have been used to examine insight, and the Mood Disorders Insight Scale (MDIS) was modified specifically for patients with mood disorders (13–15). Impaired insight has been commonly observed in many mental disorders, but most investigations have been conducted for patients with schizophrenia and bipolar disorder (16). Impaired insight has also been found among patients with MDD. Yen et al. (2005) revealed that in depressed individuals, impaired insight into or an awareness of their illness, an attribution of symptoms, and the need for treatment were 36.8, 37.2, and 15.8%, respectively (14).

Lack of insight has been associated with undesirable clinical outcomes (17) and poor neurocognition (18). It has long been recognized as a potent barrier to treatment adherence, which is associated with poorer clinical and functional outcomes (19). Lack of recognition and mistrust in mental illness treatment were considered barriers to seeking professional mental health services for depression (20). The poor insight was associated with frontal and prefrontal activities dysfunction in schizophrenia (15). In addition, impaired insight has been suggested to be associated with executive dysfunction, including working memory, speed of processing, reasoning, and problem-solving (18). Poor insight has also been related to difficulties in cognition flexibility based on visual discrimination and attentional set formation maintenance (21).

Cognitive impairment related to deficits in executive function is clinically significant, which may result in occupational difficulties and unemployment in patients with depression (22, 23). Cognitive recovery can improve overall outcomes (24). However, heterogeneity regarding cognitive impairment has been mentioned in patients with MDD, in particular executive functions (25, 26). One study revealed that some medication-free depressed individuals showed significant reductions in executive function (27), whereas others showed subtle or no executive function deficits (26, 28). Symptom severity, the number of previous depressive episodes, a higher level of resistance to antidepressant strategies, level of education, and age may, to some extent, explain the heterogenic cognitive profile (29, 30). Nonetheless, to our knowledge, factors associated with impaired executive function remain inconsistent. As neuroimaging data showed that executive function was associated with the frontal prefrontal cortex (31). Thus, it would be reasonable to think that there are other factors, such as insight, which are associated with the cognitive function of depression, medicated by the frontal cortex (15, 27).

Little attention has been paid to the relationship between insight and clinical factors such as depressive severity, executive function, or outcomes of patients with MDD, while a range of studies on insight have been performed in relation to several mental disorders, especially schizophrenia and bipolar disorder. The objective of this study is to examine the relationship of insight to executive function and recurrence in MDD patients in a longitudinal follow-up cohort, and to systematically examine the role of insight and other clinical factors in the risk of recurrence.

## 2. Methods

### 2.1. Participants

A total of 277 patients with confirmed MDD were recruited from the Mental Health Center of the West China Hospital of Sichuan University. MDD was diagnosed using the Structured Clinical Interview based on the Diagnostic and Statistical Manual of Mental Disorders (DSM)-IV criteria by qualified psychiatrists. All were Han Chinese, aged 16–60 years, never treated with electrotherapy, never medicated by any antidepressants, antipsychotics, mood stabilizers, benzodiazepines, or other drugs affecting the central nervous system, or stopped using medicine at least 3 months prior to enrollment. For all participants, the exclusion criteria were any neurological history, loss of consciousness, pregnancy, current comorbidity, and other DSM-IV Axis I diagnosis.

### 2.2. Assessments

A longitudinal study design was employed in this study. Demographic and clinical symptom-related data were collected at baseline, including age, sex, years of education, age of onset, first episode or relapse, untreated duration, total duration, and executive function. All participants were contacted at least 1 year after initial recruitment *via* telephone and invited to participate in the follow-up assessment. “Recurrence” was defined as the appearance of a new episode after at least 2 months of recovery (32, 33). Information about depressive episodes was collected over the duration of the treatment, and recurrence-related information was collected during follow-up sessions. In total, 141 participants attended the follow-up visits.

The 17-item HAM-D was used to evaluate the severity of depressive symptoms and five symptom dimensions, including anxiety/somatization, weight, cognition, retardation, and sleep (34). According to a cross-sectional study (12), insight was assessed with the HAM-D, through the authors’ transformation of the depression insight item: 0 represents “acknowledges being depressed;” 1 represents “acknowledges illness but attributes the cause to bad food, climate, overwork, need for rest, etc.;” and 2 represents “denies being ill.” In this study, the score 0 meant “with insight;” 1 and 2 meant “without insight.”

Executive function was examined by IED task from CANTAB, which was previously revealed to be sensitive to executive dysfunction in MDD (27, 31). The IED test is a measure of rule acquisition and reversal, which mirrors the Wisconsin Card Sorting Task (WCST) (35). The IED task accessed an individual’s ability to focus on the specific compound stimuli, and to shift attention as required, which response inhibition, shifting, and cognitive flexibility. Two patterns composed of color-filled shapes and/or white lines appeared on the computer screen each time. Participants were required to select the correct one (six consecutive correct responses). If at any stage, the participant failed to reach this criterion after 50 trials, the test terminated. Participants were required to maintain their attention to different stimuli examples in the same dimension and then shift attention to the previously irrelevant dimension. Nine stages were applied. There were some main outcomes of the IED test: pre-ED errors, EDS errors, total errors, completed stage errors, stages completed, total trials, and completed stage trials. Except for stages completed, the other indicators were lower is better.

The study was approved by the Ethics Committee of West China Hospital, Sichuan University, and all participants provided written informed consent.

### 2.3. Statistical analyzes

The participants were classified dichotomously as with or without insight at baseline and grouped by recurrence or non-recurrence at follow-up. The analysis was conducted in two stages.

First, the differences between the two groups based on their socio-demographic and clinical characteristics were evaluated using Chi-square tests for categorical variables and independent sample *t*-tests for continuous variables.

Next, binary logistic regression analysis (forward: conditional) was performed to explore the relationships between measures of socio-demographic and clinical characteristics, cognition function, and insight at baseline. In this model, the dependent variable was insight, and the independent variables were patients’ clinical and socio-demographic variables, which were significantly different between the groups with and without insight. Similarly, the predictors of depression recurrence at follow-up were calculated using binary logistic regression analysis. Sex, age, and years of education were covariates in these analyzes.

Statistical analyzes were performed using SPSS 26.0 0 (IBM Corp., Armonk, NY, United States). The two-tailed level of statistical significance was set at *p* < 0.05.

## 3. Results

### 3.1. Differences in demographics and clinical characteristics of patients with MDD grouped by insight and recurrence

In this study, 277 MDD patients were recruited (Table 1), of whom 59 (21.3%) were without insight. Patients without insight had significantly higher total scores and factor scores (anxiety/somatization, weight, retardation, and sleep) of HAM-D than those with insight (all *p* < 0.05). A later onset age was more prevalent in patients without insight than in those with insight (*t* = 2.138, *p* = 0.03). There were no significant differences in terms of age, sex, years of education, total duration of depression, first episode/relapse, and untreated duration between those who could judge and realize their mental status and those who could not. Compared to patients with MDD who had insight, those without insight made more mistakes prior to the extra-dimensional shift of the task (*t* = 4.784, *p* = 0.03) and on stages successfully completed (*t* = 4.922, *p* = 0.019). Furthermore, the number of trials undertaken on all successfully completed stages differed between the groups (*t* = 5.53, *p* = 0.019), which indicates that MDD patients without insight demonstrated worse cognition function.

**Table 1:** Differences in demographics and clinical characteristics of patients with MDD grouped by insight and recurrence.

Item	Without insight (*N* = 59)	With insight (*N* = 218)	*t*/*χ*^2^	Non-recurrence (*N* = 99)	Recurrence (*N* = 42)	*t*/*χ*^2^
Demographic variables						
Age	29.88 ± 10.207	27.86 ± 10.709	1.3	27.34 ± 11.309	28.07 ± 8.414	−0.375
Sex (M/F)	17/42	72/146	0.378	35/64	15/27	0.002
Years of education	13.98 ± 3.104	13.29 ± 3.275	1.45	13.28 ± 3.245	14.24 ± 2.721	−1.673
Clinical characteristics						
Age of onset	27.53 ± 8.728	24.63 ± 9.242	2.16^*^	24.38 ± 10.097	25.66 ± 7.738	−0.733
Untreated duration	17.32 ± 30.438	14.15 ± 19.578	0.969	14.37 ± 14.944	18.45 ± 37.719	−0.924
First episode (Y/N)	39/20	143/75	0.005	65/34	25/17	0.480
Total duration (month)	31.50 ± 51.717	29.40 ± 42.442	0.321	25.19 ± 37.632	29.59 ± 47.491	−0.573
HAM-D total scores	23.86 ± 5.128	19.59 ± 5.736	5.187^***^	19.00 ± 5.883	22.64 ± 4.611	−3.572^***^
Anxiety/Somatization factor score	5.66 ± 1.748	3.77 ± 1.828	7.129^***^	3.90 ± 1.903	4.95 ± 1.847	−3.032^*^
Weight factor score	1.36 ± 0.846	1.00 ± 0.895	2.740^*^	0.92 ± 0.841	1.19 ± 0.890	−1.721
Cognition factor score	3.76 ± 1.546	3.57 ± 1.767	0.767	3.48 ± 1.897	3.93 ± 1.614	−1.325
Retardation factor score	7.92 ± 1.774	6.93 ± 2.141	3.256^*^	6.63 ± 2.033	8.05 ± 1.738	−3.956^***^
Sleep factor score	4.15 ± 1.595	3.52 ± 1.704	2.551^*^	3.32 ± 1.701	3.48 ± 1.627	−0.495
Insight (Y/N)				84/15	27/15	7.444^*^
IED task						
Pre-ED errors^a^	10.04 ± 7.912	8.08 ± 5.317	4.784^*^	8.38 ± 5.490	8.30 ± 6.374	0.001
ED shift errors^a^	10.41 ± 9.193	10.94 ± 9.998	0.175	10.24 ± 9.437	9.96 ± 8.573	0.005
Stages completed^b^	8.44 ± 0.939	8.24 ± 1.322	1.395	8.47 ± 1.005	8.30 ± 1.145	0.636
Total errors^a^	24.26 ± 11.872	22.70 ± 12.493	0.609	22.63 ± 12.902	21.82 ± 11.332	0.01
Completed stage errors^a^	16.93 ± 10.131	13.65 ± 9.342	5.530^*^	15.43 ± 10.173	13.81 ± 7.817	0.341
Total trials^a^	94.22 ± 20.963	89.95 ± 21.628	1.861	90.48 ± 22.259	89.60 ± 20.210	0.019
Completed stage trials^a^	79.53 ± 21.016	72.67 ± 21.940	4.922^*^	77.01 ± 21.560	73.34 ± 19.147	0.386

MDD, major depressive disorder; HAM-D, Hamilton Depression Rating Scale; IED, Intra/extra-dimensional shift task; ^a^Lower is better; ^b^Higher is better; ^*^p < 0.05; ^***^p < 0.001.

A total of 141 participants with depression completed the follow-up in 1–5 years, and 42 patients (29.8%) experienced recurrence. We also compared the demographics and clinical characteristics between those follow-ups and lost-to-follow at baseline, there were no statistical differences in the two groups except for sleep disturbance scores (*t* = −2.914, *p* = 0.004; see Supplementary Table S1). Patients who experienced recurrence were mainly those without insight (*χ*^2^ = 7.444, *p* = 0.007) and with a higher total (*t* = 3.572, *p* < 0.001) and factor scores, including anxiety/somatization (*t* = 3.302, *p* = 0.003) and retardation (*t* = 3.956, *p* < 0.001) in HAM-D, compared to those without recurrence.

### 3.2. Correlative factors of insight

The variables in this model included onset age, anxiety/somatization scores, weight scores, retardation scores, and sleep scores of HAM-D, Pre-ED errors, completed stage errors, and completed stage trials. As shown in Table 2, the binary logistic regression identified two factors after controlling for sex, age, and education years: anxiety (OR: 0.564, *p* < 0.001) and IED completed stage errors (OR: 0.968, *p* = 0.040) that related to insight.

**Table 2:** Correlative factors of insight in binary logistic regression model.

Insight	95% CI		^a^OR	B	*p*
	Lower	Upper			
Anxiety/Somatization factor score	0.467	0.681	0.564	−0.573	<0.001
Completed stage errors	0.938	0.998	0.968	−0.033	0.040

95% CI = 95% confidence interval; ^a^OR = adjusted odds ratio which was based on binary logistic regression (forward: conditional) of insight (with vs. without) as a dependent variable, sex and age, education years, onset age, anxiety/somatization scores, weight scores, retardation scores, and sleep scores of HAM-D, Pre-ED errors, completed stage errors, and completed stage trials as independent variables.

### 3.3. Correlative factors to predict depression recurrence

The variables in this model included anxiety/somatization scores and retardation scores of HAM-D, and insight. The binary logistic regression revealed two factors after controlling for sex, age, and education years, including insight (OR: 2.557, *p* = 0.037) and retardation (OR: 1.445, *p* = 0.001) that predicted recurrence (Table 3). The results demonstrated that patients with MDD without insight and more serious retardation symptoms tend to have recurrences.

**Table 3:** Binary logistic regression model to predict recurrence.

Recurrence	95% CI		^a^OR	B	*p*
	Lower	Upper			
Insight	1.06	6.168	2.557	−0.368	0.037
Retardation factor score	1.162	1.797	1.445	0.939	0.001

95% CI = 95% confidence interval; ^a^OR = adjusted odds ratio which was based on binary logistic regression (forward: conditional) of recurrence (yes vs. no) as a dependent variable, sex and age, education years, anxiety/somatization scores and retardation scores of HAM-D, and insight as independent variables.

## 4. Discussion

While only a few researches have previously addressed the relationship between MDD and insight, the present study constitutes an attempt to explore such an association and found that lack of insight is associated with severe depressive symptoms, poor cognitive flexibility, and a high risk of recurrence in patients with MDD.

Our findings showed that patients with MDD without insight suffered more severe depressive symptoms than those with insight. This result was in line with previous research, which revealed that poor insight was associated with severe depressive symptoms in patients with obsessive–compulsive disorder (35). On the contrary, Yen et al. (2005) discovered that patients with more severe depression have better insight into and awareness of the disorder (14). Extensive data have established that the nature of insight differs across mental disorders, and the neuropsychological mechanisms in patients with MDD may differ from others (36). Another possible explanation for this difference is that different dimensions of insight have different effects on the severity of depressive symptoms. Besides, the unawareness of mental disorder is often not reported, the manifestations and course of the disease, and unawareness of treatment may lead to high rates of treatment non-adherence, which result in more severe depressive symptoms (17, 37).

The findings of this study have some clinical implications. Patients with MDD who complain less about depression as emotional disturbances but more about physical discomforts may need more attention, as they may be without insight into the illness. Previous studies have shown that anxious depression is associated with more serious depressive symptoms and poorer acute treatment outcomes than non-anxious depression (38, 39). Moreover, an anxious distress specifier predicted a greater frequency of experiencing antidepressant side effects at the 2-year follow-up (40). It is possible that patients with anxiety features were more sensitive to somatic changes during antidepressant treatment and may have been more likely to drop out of treatment due to side effects (40). Future studies need to divide patients with depression into subgroups of different insight types to verify this result.

It is demonstrated that the onset of depression at an earlier age was correlated with better insight, in agreement with a previous study on early psychosis (41). Perhaps with the continuous popularization of mental health education in China, the younger generation has increased their awareness of mental health services (42). Therefore, it is easier for young people to understand the symptoms of depression and to seek treatment (43). However, no significant relationship between onset age and insight was observed in the binary logistic regression model, including depression severity and cognitive function. Due to the lack of available data, more research is needed to illustrate the relationship between onset age and insight in patients with depression.

It was also noted that impaired performance in the IED task is related to poorer insight, suggesting that poor insight in individuals with depression is also correlated with neurocognitive impairment, mainly in cognitive flexibility. This association between poor insight and cognitive flexibility disturbance may be medicated by frontal cortical function (15). To the best of our knowledge, no study has investigated the relationship between levels of insight and neurocognitive profiles in patients with MDD. This is in accordance with an analysis indicating that poor insight is related to executive dysfunction in schizophrenia (21). In addition, similar to the study, neurocognitive dysfunction, specifically failure to change set-shifting and monitor error responses, leads to poor insight in psychosis (44). In addition, compared to those with typical intellectual levels, those with a premorbid intelligence quotient (IQ) greater than 120 showed better clinical insight (45). A study performed by path analyzes reported that neurocognition influenced insight, which then influenced psychosocial function (46). This is also consistent with other studies that have indicated that good cognitive function may be a basic condition for clinical insight (47). As is well known, the association intensity varies from insight domains to types of neurocognition (48). Further studies are required to provide evidence to support the relationship between insight and neurocognition in patients with MDD.

Consistent with earlier studies, we found that lack of insight in patients with MDD is associated with a greater likelihood of depressive episode recurrence (49). On the one hand, those who lack insight have problems with service engagement and compliance with medication (49). On the other hand, poor therapeutic alliance and poor insight are predictors of non-adherence among patients with mood disorders (50). It is obvious that lower adherence to treatment increases the risk of recurrence observed by clinicians (51).

In addition, our findings support the results of some previous studies, which found that more severe depressive symptoms lead to recurrence (4). Retardation, a core symptom of depression, can independently predict the recurrence of the disease. The severity of the depressive episode has always been associated with a higher likelihood of recurrence among adults with MDD (6). Thus, clinicians should use these clinical features as indicators of future recurrence.

Some limitations of the study should be noted. Firstly, the measure of insight is focused on global insight. The comprehensive tools to access several dimensions of insight need to be employed, such as the MDIS, in order to formulate targeted treatment and recurrence prevention strategies in future studies. Secondly, this is only an exploratory study on the correlation between lack of insight and neurocognitive impairment in patients with MDD. Further research is needed to discuss whether they have common pathological mechanisms and how to intervene in these mechanisms to reduce recurrence. Finally, as patients with insight may have better treatment compliance through follow-up, it limits the statistical power to find the relationship between insight and the recurrence of depressive episodes in individuals with MDD in the real world. This study did not find any distinctions between those who participated in follow-up and those who did not in terms of insight and other characteristics. However, to bolster the statistical power of the results, a greater follow-up rate is necessary for future research.

## 5. Conclusion

In summary, this study provides evidence that MDD patients without insight present more severe depressive symptoms, and a high risk to recur. Therefore, this study reminds us that the insight of MDD also needs to be evaluated in detail in order to improve MDD patients’ long-term efficacy.

## Data availability statement

The data are available upon request to the corresponding author, Prof. Xiaohong Ma (maxiaohong@scu.edu.cn).

## Ethics statement

The studies involving human participants were reviewed and approved by Ethics Committee of West China Hospital, Sichuan University. Written informed consent to participate in this study was provided by the participants’ legal guardian/next of kin.

## Author contributions

MW: conceptualization, investigation, data curation, methodology, formal analysis, writing–original draft, and visualization. QL: investigation, data curation, and writing–original draft. XY: conceptualization, methodology, formal analysis, writing–review and editing, supervision, and project administration. YD, YW, ZZ, and RL: investigation and data curation. YM: investigation. QW, and TL: supervision and project administration. XM: resources, writing–review and editing, supervision, and project administration. All authors have approved the final version of the manuscript.

## Funding

This study was supported by the Key research and Development Program of Science and Technology Department of Sichuan Province (Nos. 2022YFS0346, 2022YFS0348), the Program of Chengdu Science and technology (No. 2021-YF05-00272-SN), National Natural Science Foundation of China (No. 82001432), China Postdoctoral Science Foundation (Nos. 2020TQ0213, 2020 M683319), the Open Project Program of the National Laboratory of Pattern Recognition (No. 202000034), and West China Hospital Postdoctoral Science Foundation (No. 2020HXBH104).

## Conflict of interest

The authors declare that the research was conducted in the absence of any commercial or financial relationships that could be construed as a potential conflict of interest.

## Publisher’s note

All claims expressed in this article are solely those of the authors and do not necessarily represent those of their affiliated organizations, or those of the publisher, the editors and the reviewers. Any product that may be evaluated in this article, or claim that may be made by its manufacturer, is not guaranteed or endorsed by the publisher.
