# Supplementary material for: Relationship of insight to neurocognitive function and risk of recurrence in depression: A naturalistic follow-up study
Source: Front Psychiatry. 2023 Mar 16;14:1084993. doi: 10.3389/fpsyt.2023.1084993 (PMC10060510; doi:10.3389/fpsyt.2023.1084993)
Supplement: Supplementary file 1 [file Table_1.DOCX]

**sTable 1. Differences in demographics and clinical characteristics of patients with MDD grouped by follow-up**

| **Item** | **Lost to follow-up (N=136)** | **Follow-up**  **(N=141)** | **t/χ^2^** | **p** |
| --- | --- | --- | --- | --- |
|  |  |  |  |  |
| **Demographic variables** | | | | |
| Age | 29.04±10.72 | 27.56±10.506 | -1.163 | 0.246 |
| Sex (M/F) | 39/97 | 50/91 | 1.461 | 0.140 |
| Years of education | 13.31±3.379 | 13.57±3.12 | 0.662 | 0.509 |
| **Clinical characteristics** | | | | |
| Age of onset | 25.76±8.935 | 24.76±9.447 | -0.897 | 0.371 |
| Untreated duration | 14.03±20.458 | 15.59±24.01 | 0.578 | 0.564 |
| First episode(Y/N) | 97/39 | 91/50 | 1.461 | 0.140 |
| Total duration (month) | 33.09±48.858 | 26.71±39.744 | -1.194 | 0.234 |
| HAM-D total scores | 20.93±5.967 | 20.09±5.766 | -1.204 | 0.230 |
| Anxiety/Somatization factor score | 4.12±2.002 | 4.21±1.941 | 0.37 | 0.711 |
| Weight factor score | 1.15±0.926 | 1±0.862 | -1.437 | 0.152 |
| Cognition factor score | 3.6±1.616 | 3.62±1.823 | 0.068 | 0.946 |
| Retardation factor score | 7.23±2.164 | 7.05±2.051 | -0.704 | 0.482 |
| Sleep factor score | 3.96±1.677 | 3.37±1.675 | -2.914 | 0.004 |
| Insight (Y/N) | 107/29 | 111/30 | 0.001 | 0.554 |
| **IED task** | | | | |
| Pre-ED errors ^a^ | 8.64±6.278 | 8.36±5.744 | -0.385 | 0.701 |
| ED shift errors ^a^ | 11.52±10.446 | 10.16±9.158 | -1.158 | 0.248 |
| Stages completed ^b^ | 8.14±1.423 | 8.42±1.048 | 1.862 | 0.064 |
| Total errors ^a^ | 23.7±12.305 | 22.39±12.421 | -0.885 | 0.377 |
| Completed stage errors ^a^ | 13.74±9.647 | 14.94±9.534 | 1.048 | 0.296 |
| Total trials ^a^ | 91.53±21.499 | 90.22±21.601 | -0.506 | 0.614 |
| Completed stage trials ^a^ | 72.29±22.83 | 75.91±20.871 | 1.381 | 0.168 |

Note: MDD = major depressive disorder; HAM-D = Hamilton Depression Rating Scale; IED = Intra/extra- dimensional shift task; ^a^ Lower is better.
